# Validity and Reliability of the eHealth Analysis and Steering Instrument

**DOI:** 10.2196/med20.2571

**Published:** 2013-08-22

**Authors:** Olivier A Blanson Henkemans, Elise ML Dusseldorp, Jolanda FEM Keijsers, Judith M Kessens, Mark A Neerincx, Wilma Otten

**Affiliations:** ^1^Nederlandse Organisatie voor Toegepast Natuurwetenschappelijk Onderzoek (TNO), Department LifestyleLeidenNetherlands

**Keywords:** self-care, psychometrics, validity, reliability, scale analysis, effectiveness, self-management support

## Abstract

**Background:**

eHealth services can contribute to individuals’ self-management, that is, performing lifestyle-related activities and decision making, to maintain a good health, or to mitigate the effect of an (chronic) illness on their health. But how effective are these services? Conducting a randomized controlled trial (RCT) is the golden standard to answer such a question, but takes extensive time and effort. The eHealth Analysis and Steering Instrument (eASI) offers a quick, but not dirty alternative. The eASI surveys how eHealth services score on 3 dimensions (ie, utility, usability, and content) and 12 underlying categories (ie, insight in health condition, self-management decision making, performance of self-management, involving the social environment, interaction, personalization, persuasion, description of health issue, factors of influence, goal of eHealth service, implementation, and evidence). However, there are no data on its validity and reliability.

**Objective:**

The objective of our study was to assess the construct and predictive validity and interrater reliability of the eASI.

**Methods:**

We found 16 eHealth services supporting self-management published in the literature, whose effectiveness was evaluated in an RCT and the service itself was available for rating. Participants (N=16) rated these services with the eASI. We analyzed the correlation of eASI items with the underlying three dimensions (construct validity), the correlation between the eASI score and the eHealth services’ effect size observed in the RCT (predictive validity), and the interrater agreement.

**Results:**

Three items did not fit with the other items and dimensions and were removed from the eASI; 4 items were replaced from the utility to the content dimension. The interrater reliabilities of the dimensions and the total score were moderate (total, κ=.53, and content, κ=.55) and substantial (utility, κ=.69, and usability, κ=.63). The adjusted eASI explained variance in the eHealth services’ effect sizes (*R^2^*=.31, *P*<.001), as did the dimensions utility (*R^2^*=.49, *P*<.001) and usability (*R^2^*=.18, *P*=.021). Usability explained variance in the effect size on health outcomes (*R^2^*=.13, *P*=.028).

**Conclusions:**

After removing 3 items and replacing 4 items to another dimension, the eASI (3 dimensions, 11 categories, and 32 items) has a good construct validity and predictive validity. The eASI scales are moderately to highly reliable. Accordingly, the eASI can predict how effective an eHealth service is in regard to supporting self-management. Due to a small pool of available eHealth services, it is advised to reevaluate the eASI in the future with more services.

## Introduction

### Background

eHealth services, contributing to self-management, are developed and implemented on a daily basis. The Internet is flooded with websites and apps, which offer support for individuals to perform lifestyle-related activities and decision making, to maintain a good health, or to mitigate the effect of an (chronic) illness on their health. For example, Apple offers more than 200 apps, which provide information about healthy habits, offer the possibility to keep a diet, help monitoring physical activity, and facilitate managing an illness, such as diabetes. These websites and apps all claim that they can help to maintain a healthy lifestyle and contribute to a person’s health. But how effective are these eHealth services?

Various randomized controlled trials (RCTs) examined the effectiveness of eHealth services on self-management, with a large variety in effectiveness. For example, Norman et al. reported heterogeneity of studies with respect to participants, type of intervention and outcomes, and mixed findings related to the outcome [1]. As a result, it is difficult to generalize these findings to all eHealth services supporting self-management. In addition, many new health services are developed and should the effectiveness of each of these be examined empirically in an RCT?

Conducting an RCT takes extensive time and effort. Enrolling and studying people using an eHealth service for a longer period of time to examine its effectiveness may take a year or more. In addition, one has to deal with high levels of attrition when people use eHealth services [2]. Meanwhile, when the results are published, general knowledge and technological developments about eHealth are already a number of steps ahead [3]. Although considered the “gold standard” in empirical research on medical interventions, these RCTs are not an efficient way to answer our question how effective an eHealth service is at this time. Moreover, when evaluating eHealth services it is suggested to apply “methodological pluralism” (ie, undertaking combined quantitative and qualitative work) [4] and to examine changes and effects of using the eHealth service on various levels, such as the micro-level (eg, user health service), meso-level (eg, health organization), and macro-level (eg, society) [5]. Accordingly, there is a need for a rating instrument which can be used efficiently, provides an agenda to discuss how an eHealth service can contribute to self-management, and finally which is valid and reliable to provide a forecast on the effectiveness of an eHealth service on self-management, that is, an instrument which collects data “quick, but not dirty”.

The present literature does not provide such an instrument. Most instruments are concerned with rating the quality of the content of health websites (eg, Health Website Rating Instrument, HWRI [6] and for an overview see [7]), standards to report studies on eHealth devices (eg, Consolidated Standards of Reporting Trials of Electronic and Mobile HEalth Applications and online TeleHealth, CONSORT-EHEALTH [8]), or toolkits to promote the implementation of eHealth (eg, eHealth implementation toolkit, e-hit [9]). However, we need an instrument that not only evaluates the quality of the content of a website, description of the study, or implementation of the service, but that judges if the eHealth device effectively supports changing health-related behavior (ie, se-management).

### eHealth Analysis and Steering Instrument: Dimensions and Categories

The eHealth Analysis and Steering Instrument (eASI) is developed to measure the expected effectiveness of eHealth services on self-management, without necessitating the endeavors of an RCT or more formative research on various levels (ie, micro-, meso-, and macro-level). The eASI is based on a literature review, examining definitions and operationalization of the effectiveness of eHealth [10]. This review covered the literature on health promotion, self-management and self-regulation, human-computer interaction, usability, and the development and implementation of health-promoting interventions, including interactive health technologies (ie, eHealth) [11-20]. The review elicited various techniques and strategies contributing to the effectiveness of health innovations. Examples are providing feedback to create health awareness, offering decision aids, and goal setting. In addition, it elicited usability aspects contributing to the effectiveness of technology in general. Only one paper looked at evaluation of usability in eHealth services. In this paper, the usability guidelines, as originally introduced by Norman and Nielsen, are used as principal evaluation items, because no new evaluation items have been specifically developed for testing interactive health technologies. The guidelines for usability include interface consistency, error prevention, and tailoring to user characteristics. Finally, the review elicited aspects related to the content of the technological health-promoting intervention, which contribute to its effectiveness. Here, aspects cover analyzing the health problem, identifying causes of the health problem and the extent to which the intervention attends to these factors, and the constituency for the intervention.

These resulting aspects were integrated in a conceptual framework consisting of 3 dimensions, contributing to the effectiveness of eHealth supporting self-management. These dimensions are: (1) utility, a scale of how functional the service is (ie, what is self-management and how is it operationalized in the rated eHealth services), (2) usability, a scale of how usable the service is (ie, how easy and enjoyable is it to perform self-management with this service), and (3) content, a scale of the quality of the content of the service (ie, does this service contain content, which succeeds in convincing why it is important for the user to perform self-management.).

These dimensions were operationalized in 3 subscales by formulating Likert-type items. The dimensions contain different categories, which in turn cover 43 items, which are rated dichotomously.

The face validity of this 43-item version of the eASI was evaluated by a group of Dutch experts (n=28) in a Delphi procedure [21]. Through this Delphi study, we reached consensus that 35 items were considered relevant for measuring the effectiveness of eHealth (see Table 1). The 35 items are divided across 12 categories, which in turn are divided across the three dimensions: utility, usability, and content. For an overview of the items, see Multimedia Appendix 1.

The eASI is developed for intermediates, such as health care insurance companies, health care givers, and eHealth developers. This target group can directly act based on the eASI outcomes. They can reimburse, buy and apply services, or determine how to (re)develop them. A first application of the eASI showed that it can be used to analyze the expected effectiveness of eHealth services and provide steering for improvement [10]. However, there are no data on its validity and reliability. Therefore, our study has 3 aims to address these issues: First, the construct validity: the degree to which the scores of eASI are consistent with our hypotheses, regarding internal relationships between items within the different dimensions—utility, usability, and content [22]. Second, the interrater reliability: the degree of agreement among the raters for each item of the eASI, the total score on the eASI, and the three dimensions [22]. Third, the predictive validity: the degree to which the scores on eASI (ie, total score and dimensions) predict the effect sizes of the rated eHealth services observed in RCTs [23].

**Table 1:** Dimensions and categories defined in the eASI and the number of items they contain.

Dimension	Categories	Number of items
Utility	Insight in health condition	3
	Self-management decision making	3
	Performance of self-management	4
	Involving the social environment	4
Usability	Interaction	4
	Personalization	3
	Persuasion	4
Content	Description of health issue	2
	Factors of influence	2
	Goal of eHealth service	3
	Implementation	2
	Evidence	1
Total		35

## Methods

### Focus

To examine the validity and reliability of the eASI, various eHealth services needed to be rated using the eASI. These ratings served to examine the construct validity and interrater reliability. In order to study the predictive validity of the eASI, the effectiveness of these eHealth services had to be assessed in an RCT. Although the RCT is sometimes criticized as too limited to assess the effectiveness of eHealth services [4,5], we consider the RCT as a suitable and conservative approach to examine the effects of stand-alone eHealth services to support individual users in their self-management. To demonstrate the predictive validity, the effect sizes of the eHealth services found in an RCT needed to be compared with the eASI rating result of that eHealth service.

### Selection of eHealth Services

Systematic literature searches in electronic databases (Pubmed, MEDLINE, CINAHL, and PsycInfo) were conducted for RCTs of eHealth services, which aimed at increasing self-management. We used the search phrase (online OR Internet OR eHealth) AND (self-management OR self-care OR health-promotion) AND (randomized controlled trial OR RCT) as title and abstract words or MeSH terms. Article reference lists were examined for additional papers. A total of 14,531 papers were identified.

Subsequently, titles and abstracts of the papers were screened using the following criteria: First, the RCT evaluated an eHealth service (ie, online or Web-based or Internet-based therapy, treatment, or intervention) and the outcome measure was self-management behavior (ie, behavior conducted by the user to improve or maintain health or minimize impact of illness on health). Second, the results of the full trial were published or in press. This screening elicited 64 studies. Finally, we screened if the studied eHealth service used the Dutch, English, French, or German language and was available to be rated by the eASI in our study. This screening elicited 16 services (see Table 2).

**Table 2:** Overview of the eHealth service and RCT evaluation (N=16).

eHealth service (country)	Study	Service characteristics^a^
1. Drinktest (Netherlands)	Boon et al [24]	Problem drinkers
		Assessment and advice
		SA
		Reduce alcohol consumption
2. Moodgym (Australia)	Powell et al [25]	People with (early signs of) a depression
		Web-based cognitive behavioral therapy (CBT)
		SA
		Reduce depression and anxiety
3. Interapy (Netherlands)	Ruwaard et al [26]	People with a depression
		Online assessment, diagnosis by phone and Web-based CBT
		BC
		Reduce symptoms of depression and anxiety
4. Gripp (Netherlands)	Genugten et al [27]	People who are overweight
		Web-based modular treatment focusing on goal setting, self-monitoring, and feedback
		SA
		Reduce weight gain
5. Alcoholdebaas (Netherlands)	Postel et al [28]	Problem drinkers
		Asynchronous communication with therapist, health information, and forum
		BC
		Reduce alcohol consumption
6. Diep (Netherlands)	Heinrich et al [29]	People with diabetes
		Interactive information on diabetes
		SA
		Improve diabetes regulation
7. Diabetergestemd (Netherlands)	Bastelaar [30]	People with diabetes and depression
		Web-based, guided self-help program based on CBT
		BC
		Reduce depressive symptoms
8. Fitnet (Netherlands)	Nijhof et al [31]	Teenagers with chronic fatigue syndrome
		Web-based CBT
		BC
		Improve school presence and physical functioning and reduce fatigue
9. Gezondgewichtassistent (Netherlands)	Kelders et al [32]	People who are overweight
		Website to set and achieve personal health goals and tailored health information
		SA
		Maintaining a healthy lifestyle and improve body mass index (BMI)
10. Kleurjeleven (Netherlands)	Graaf et al [33]	People with (early signs of) a depression and anxiety
		Web-based CBT
		BC
		Reduce symptoms of depression and anxiety
11. 113online (Netherlands)	Spijker et al [34]	People with suicidal ideations
		Online services, covering self-test and consultation through chat, phone and email, forum, and self-help course
		BC
		Reduce suicidal ideations
12. Patientcoach (Netherlands)	van der Meer et al [35]	People with chronic obstructive pulmonary disease (COPD) and asthma
		Web-based application for health information, self-monitoring, and eConsult
		BC
		Improve COPD and asthma regulation
13. Diabeter (Netherlands)	Blanson Henkemans et al [36]	People who are overweight
		Online lifestyle diary, setting personal goals and feedback from an avatar
		SA
		Maintaining a healthy lifestyle and improve BMI
14. Minderdrinken.nl (Netherlands)	Riper et al [37]	Problem drinkers
		Web-based CBT
		SA
		Reduce alcohol consumption
15. Alles onder controle (Netherlands)	Warmerdam et al [38]	People with (early signs of) a depression and anxiety
		Web-based CBT
		BC
		Reduce symptoms of depression and anxiety
16. Active online (Switzerland)	Wanner et al [39]	People who want to increase physical exercise
		Individually tailored counseling and motivational feedback
		SA
		Improved physical exercise

^a^Target group, intervention description, stand-alone (SA) or blended care (BC), and goals.

### Rating eHealth Services With eASI

#### Population

The eASI target user group consists of health care insurance employees in charge of acquiring eHealth services, health care givers applying eHealth, and eHealth developers. These persons are generally highly educated and use computers and Internet daily. In our study, to fit the profile of the target group, we recruited a sample of 16 men and women, aged 20-25 years, highly educated (ie, BA or MA degree), and with above average experience with computers and Internet.

Persons were recruited through the participants’ database of the Dutch Organization for Applied Sciences (TNO) through an invitational email. Computer experience of the persons, who signed up for the study, was assessed with a computer experience survey. This survey consisted of a 5-point Likert scale, ranging from low (little computer and Internet experience) through high (extensive computer and Internet experience, including programming). All participants scored at least 4 points. Participants were invited to rate eHealth services and they received a small fee for their participation. They did not have prior experience with the eASI.

#### eASI Instrument

The eASI is based on a literature review of factors related to the effectiveness of eHealth services, regarding self-management and health outcomes [10]. For the study, we applied the eASI, which was tested on face validity and improved accordingly. The eASI contained 35 items, which were rated dichotomously (item is applicable or not applicable to eHealth service). An eHealth service could score 0-35 points in total, 0-14 points for utility, 0-11 points for usability, and 0-10 points for content. The higher the score, the more effective an eHealth service is expected to be.

#### Procedure

The rating sessions lasted approximately 2.5 hours and started with a short questionnaire assessing demographics (ie, gender, year of birth, and education level) and use of eHealth (on a 4-point scale: never, sometimes, regularly, and often). Further, the participants received a short training on how to rate with the eASI. The training covered the goal of the eASI, explanation of the three dimensions, and instructions on how to use the eASI to rate the eHealth services. These instructions were also available on paper during the rating. The rated eHealth services were presented on a PC and the eASI was filled in on paper. Finally, we surveyed how the raters experienced rating eHealth services with the eASI. The raters were surveyed after each rated service, using a 5-point Likert scale and an open question, on the experienced clarity of the items, the effort to answer them, and the ability to rate a service with the eASI. In addition, we posed an open question about the positive and negative features of the eASI.

It would be too demanding for each participant to rate all eHealth services with the eASI. Therefore, each eHealth service was rated by 3 participants. They were randomly selected from the pool of 16 participants in such a way that each of the 16 participants rated 3 eHealth services. For example, the eHealth service by Postel et al was rated by raters 1, 12, and 14. The score of each service on the eASI was calculated as follows: First, we computed the services’ total eASI score and score per dimensions, per rater (ie, sum score). Second, we averaged the three raters’ sum scores.

### Statistical Analysis

#### Construct Validity

To determine the construct validity, that is, to confirm the existence of the predefined three dimensions, we conducted confirmatory factor analysis (ie, the oblique multiple group method) [40,41]. We tested if the eASI ratings fit the hypothesized structure. For each dimension, we calculated the reliability statistic (ie, Cronbach alpha) and for each item 3 correlations: the correlation with the dimension it is assumed to belong to (with an item-rest correlation) and the correlations with the other two dimensions. If the first correlation (the item-rest correlation) was larger than the latter two, the predefined structure was confirmed.

Because we had scores from 3 raters per item, we calculated the Cronbach alpha from 3 random samples in regard to the rater (ie, we randomly selected one score per item; and this was repeated 3 times). On the basis of the results, an alternative structure of the eASI was considered.

#### Interrater Reliability

As an index of the interrater reliability, a generalized kappa was computed (ie, Light’s kappa) [42]. For the analysis, we assumed that the raters were interchangeable (ie, each of the raters could “act” as the first, second, or third rater), and we organized the data for each item accordingly. We permuted the order of the values in each row 1000 times, resulting in 1000 data sets. For each permuted data set, we computed Light’s kappa, resulting in 1000 values of kappa. As summary statistics, we used the computed mean kappa of these 1000 values, and the minimum and maximum. We used the interpretation of kappa, as listed in Table 3 [43].

#### Predictive Validity

To determine the predictive validity, we first analyzed how the RCTs measured the effectiveness of the eHealth services. Self-management behavior is influenced by personal and environmental determinants (eg, intention, attitude, and subjective norm). In turn, self-management behavior results in health outcomes. This behavioral model is based on, among others, the theory of reasoned action and the theory of planned behavior [44]. These social cognitive theories of behavior distinguish 3 elements of behavior: (1) the determinants of an individual’s behavior, (2) the intention to perform a behavior, and (3) the actual behavior itself. Many health outcomes are linked to specific behaviors, thus a fourth step that can be distinguished, which is the impact of the behavior on an individual’s health. This enabled us to categorize the measures of the different studies and compare effect sizes. First, we calculated the effect sizes (ie, Hedges *g*) of each service in regard to (1) determinants of behavior, (2) self-management behavior, and (3) health outcomes [45]. Second, we conducted a regression analysis in which we studied the relation between the eHealth services’ effect size in regard to determinants, health behavior and health outcomes, and their averaged sum scores on the eASI in total and per dimension. For example, the analysis showed that the eHealth service “Alcohol de baas” (Look at your drinking) had an effect size of 1.15 regarding self-management behavior. The sum score of the three raters on average was 31.67 on the eASI total (90% of maximum total score), 13.00 on utility (93% of maximum total score), 9.33 on usability (85% of maximum total score), and 9.33 on content (93% of maximum total score). In our regression analysis, we analyzed if eHealth services with a high effect score also had a high eASI score, just as Alcohol de baas, and *vice versa*.

#### Computational Note

The construct validity analyses were performed in SPSS (version 20.0); the predictive validity analyses were performed in Comprehensive Meta-Analyses (version 2) [46], and the interrater reliability analyses were performed using the package “psy” in the R software environment [47,48].

**Table 3:** Interpretations of kappa [43].

Kappa statistic	Strength of agreement
<.00	Poor
.00-.20	Slight
.21-.40	Fair
.41-.60	Moderate
.61-.80	Substantial
>.80	Almost perfect

## Results

### Participants

The study sample consisted of 7 male and 9 female participants, between the age of 20 and 25 years (mean 22.06, SD 1.57). They had a Bachelor (BA) or Master (MA) degree. They sometimes used eHealth services.

### Construct Validity

A first step in the construct validity is the internal consistency of the items belonging to a construct. The dimensions utility, usability, and content had a Cronbach alpha of .53,.41, and .49, respectively. An inter-item correlation analysis of items in own dimension versus items in other dimensions showed that items 5 and 35 had a negative correlation with their own dimension (−.35 and −.27, respectively) and a weak correlation with the other two dimensions. Therefore, we followed a number of steps to come to a new structure and to improve the overall inter-item correlation.

First, we discarded items 5 and 35 and redid the inter-item correlation analysis. The correlation improved, but showed that items 11-14 better correlated with the dimension content than with utility (.30 vs .06, .68 vs .49, .51 vs .04, and .12 vs −.11, respectively). Second, we discarded items 5 and 35 and placed items 11-14 in the dimension content and redid the inter-item correlation analysis. The result was that item 30 had a negative correlation with its own dimension (−33). Third, we discarded item 30 and redid the inter-item correlation analysis. Internal consistency statistics of the new version of eASI with 32 items, with items 5, 30, and 35 discarded and items 11-14 placed in the dimension content, were as follows. The dimensions utility, usability, and content had a Cronbach alpha of .61, .56, and .62, respectively. This new and final version is listed in Multimedia Appendix 1.

### Interrater Reliability

The interrater reliability of most items was moderate to almost perfect (κ>.41 and κ>.81, respectively), except for the following 6 items: 14, 15, 17, 28, 29, and 31. For 3 items (16, 25, and 30), Light’s kappa could not be computed, because there was no variability in the scores among the raters. All raters scored a “1” (ie, yes) on these eASI items.

The interrater reliabilities of the dimensions and the total score varied between moderate (total and content) and substantial (utility and usability). The interrater reliabilities of the initial structure were comparable to the ones of the new structure. The improvement of the construct validity did not go at the cost of the reliability.

### Predictive Validity

As shown in Table 4, 10 RCTs studied the effect of their eHealth service on self-management behaviors (eg, maintain diet, performing physical activity, adhering to the low-risk drinking guideline, and controlling corticosteroid). As shown in Table 5, 12 RCTs studied the effect of their eHealth service on health outcomes (ie, physical and mental health). Only 4 RCTs studied the effect of their eHealth service on determinants for self-management (eg, attitude, beliefs, knowledge, and skills). This number was too small for our predictive validity analysis. As we wanted to evaluate the eASI and not the eHealth services, we have anonymized the studies; however, services in Tables 4 and 5 are similarly denoted.

**Figure 1:**
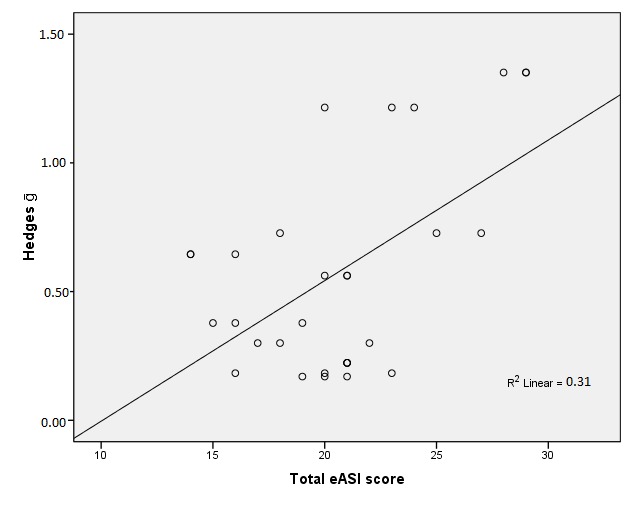
Regression of eASI total score and eHealth services’ effect size in regard to self-management behavior (Hedges g; n=10; *R ^2^*=.31; *F*_1,28_=12.56, *P*<.001).

Figure 1 shows the correlation between the eASI total score with 32 items (see Multimedia Appendix 1) and self-management behavior. The correlation was significant. The eASI total score predicted 31% of the variance in the effect sizes of the studied eHealth services (*F*_1,28_=12.56, *P*<*.*001). Furthermore, the separate eASI utility scores and eASI usability scores on self-management behavior were significant. They predicted 49% and 18% of the effect sizes (*F*_1,28_=27.37, *P*<*.*0001; *F*_1,28_=6.01, *P*=.021), respectively. The eASI content score was not significant (*R* ^2^=.05; *F*_1,28_=.54, *P*=.22).

The total score on eASI did not have a significant effect on health outcome measures (*R* ^2^=.05; *F*_1,34_=1.64, *P*=.21). Of the separate dimensions, usability (ie, new scale with 11 items) predicted 13% of the variance in the effect sizes (*F*_1,34_=5.28, *P*=.028). The other two dimensions utility and content predicted 0% and 2% variance, respectively.

**Table 4:** eHealth services’ effect sizes in RCTs of self-management behavior and sum scores on eASI total, utility, usability, and content (N=10).

eHealth services^a^	Hedges *g* (*P* value)	Score eASI total	Score eASI utility	Score eASI usability	Score eASI content
Range (min-max)	−1-1	0-32	0-9	0-11	0-12
A	.378 (.257)	19.33	7.00	4.67	6.67
B	.562 (.004)	22.00	7.00	4.33	10.33
C	.727 (.002)	22.67	8.00	7.33	7.67
D	.645 (.000)	12.33	5.00	3.67	3.67
E	.223 (.256)	22.00	8.00	6.33	7.33
F	.300 (.257)	19.67	5.00	6.00	8.33
G	.183 (.462)	19.33	4.00	7.33	8.00
H	1.151 (.000)	28.67	8.00	9.33	11.00
I	.170 (.141)	20.33	7.00	4.00	9.00
J	1.215 (.000)	21.00	7.00	7.33	6.33
Overall	.556 (.000)	20.73	6.60	6.03	7.83

^a^eHealth services have been anonymized.

**Table 5:** eHealth services’ effect sizes in RCTs of health outcomes and sum and sum scores on eASI total, utility, usability, and content (N=12).

eHealth services^a^	Hedges *g* (*P*value)	Score eASI total	Score eASI utility	Score eASI usability	Score eASI content
Range (min-max)	−1-1	0-35	0-14	0-11	0-10
A	.080 (.620)	19.33	7.00	4.67	6.67
C	.137 (.219)	22.67	8.00	7.33	7.67
E	.224 (.185)	22.00	8.00	6.33	7.33
F	.611 (.024)	19.67	5.00	6.00	8.33
G	.831 (.001)	19.33	4.00	7.33	8.00
H	.562 (.001)	28.67	8.00	9.33	11.00
J	1.194 (.000)	21.00	7.00	7.33	6.33
K	.171 (.185)	15.67	4.00	5.67	6.33
L	.541 (.000)	22.00	8.00	6.67	7.67
M	.390 (.012)	19.67	5.00	6.00	8.33
N	.227 (.515)	18.00	8.00	5.33	5.00
O	.220 (.092)	20.00	4.00	6.67	9.00
Overall	.369 (.000)	20.67	6.33	6.56	7.64

^a^eHealth services have been anonymized.

### Qualitative Evaluation of eASI

In regard to the experienced ability to rate a service with the eASI, on a scale of 1 (not at all able) through 5 (very able), the raters, on average, scored 4.06 (SD .75) after 1 rating and 3.38 (SD 1.05) after 3 ratings.

In regard to the experienced clarity of eASI, on a scale of 1 (not clear at all) through 5 (very clear), the raters, on average, scored 3.94 (SD .66) after 1 rating and 4.06 (SD .43) after 3 ratings. The items that were least clear (ie, this item was mentioned more than 6 times by the raters as not clear) were “the eHealth service aids making a decision about how to cope with a health problem in agreement with personal preferences”, “the eHealth service aids translating chosen coping strategies to a personal goal,” and “the eHealth service can be used on different platforms.”

In regard to the experienced effort to rate services with eASI, on a scale of 1 (no effort at all) through 5 (very much effort), the raters, on average, scored 2.25 (SD .66) after 1 rating and 1.94 (SD .43) after 3 ratings. The items that took most effort to rate (ie, this item was mentioned more than 6 times by the raters as difficult to rate) was “the eHealth service can be used on different platforms.”

Finally, when asked about the positive and negative features of the eASI, the raters mentioned that the eASI helped them to look at websites more accurately and systematically (n=4) and that the examples provided helped them to understand the rating items (n=3). In addition, they mentioned that it is important to bear in mind how the services is used (eg, once or continuously) (n=1) and that in some cases a caregiver is involved in the use of the service (n=2). This could affect the effectiveness. Finally, the raters suggested a rating scale instead of yes/no rating (n=3).

## Discussion

### Construct Validity

After discarding 3 items and shifting 4 items to another dimension, the three dimensions of eASI are moderately reliable (internal consistency, Cronbach alpha between .56 and .62) and the items are grouped in three distinctive dimensions. These results partly confirm our hypothetical and theory-based dimensions [10]. Accordingly, the results show that the eASI says something about the “what and how” of self-management through eHealth (utility), the ease and enjoyment using an eHealth service (usability), and why it is relevant (content).

Still, the reliability of the dimensions and especially that of content could be improved. We have two suggestions for improvement. The first suggestion is of a technical nature, namely changing the existing “Applicable/Not applicable” response scale into a 3-point rating scale. The methodological benefit of a 3-point rating scale is that there is more room for variation, which could lead to stronger correlations. The second suggestion is of a substantive nature, namely creating additional items for the content dimension or rewriting existing ones. These additional items should help discriminate the content dimension from the other two dimensions and mainly from utility, whereby the content items focus on the “why” of self-management and utility on the “what and how”. Our aim is to look for items in these two domains that are more discriminating.

### Interrater Reliability

Six items of the eASI showed a poor interrater reliability. We suggest that these items are improved in the following way. First, the formulation of the item should be made less ambiguous. In addition, the examples provided with each item should fit with the specific target group of the rated service. For example, in the case of item “Personal health data can be entered in the eHealth services (eg, BMI, blood pressure, HbA_1c_)”, the exemplary measure becomes “BMI” if the target group is overweight and “HbA_1c_” if the target group has diabetes. This requires the instrument to be adaptive. Second, the instruction for the raters should be further clarified and they could be trained. In this case, it is advisable to study if there is a learning curve and how this affects interrater reliability.

The interrater reliability could not be computed for 3 items. This finding may imply that eHealth programs in general do not vary on these items (and so the items are not informative) or that the specific sample of eHealth programs used in this study is not diverse enough. More data are needed to investigate this in more detail.

### Predictive Validity

The eASI total score predicted the impact of eHealth services on self-management behavior and health outcomes, which were assessed in RCTs. Specifically, the dimensions utility and usability were related to these effects, but content was not. These results show that the eASI is a valid instrument to predict the effectiveness of eHealth services with regard to self-management. However, the associations were small to moderately high (ie, *R* ^2^ between .05 and .31). This implies that the selection and application of eHealth services should not only be based on the eASI rating.

The total score of eASI did not predict the impact of eHealth services on health outcomes in RCTs. A possible cause is that these studies evaluated self-management among (chronically ill) patients, whereas we also looked at preventive self-management (ie, keep people healthy). It would be worth the effort to study the difference in predictive validity of the eASI for eHealth supporting healthy users or patients.

### Clarity, Ease of Use, and Considerations

The qualitative evaluation shows that the eASI scored high on clarity and ease of use. Nevertheless, there are some items, which are challenging to understand and to rate. Specifically, the item “the eHealth service can be used on different platforms” was evaluated both as unclear and challenging to rate. More and more applications are offered on mobile platforms, such as smartphones and tablet pc. These platforms have the benefit of always being at hand. Still, none of the rated services offers a mobile version (eg, app). Possibly, the services work well through mobile Internet. To rate this item, one needs to have such a platform at hand. Accordingly, as mHealth is on the rise, we feel this is an important item when rating eHealth, but also suggest reexamining the validity and reliability of this item.

The qualitative evaluation also provided some consideration in regard to how to rate eHealth services. In the rated eHealth services, we found a variation in how they are used. For example, services are used once, continuously, or in modules. In addition, some services work stand-alone, while others are part of blended care (ie, human and computerized care are alternated). To date, no study has compared these new ways of using eHealth, and they are not differentiated in the eASI. However, these aspects could very well affect the effectiveness of eHealth. Taking into account how eHealth services are operated offer direction for the possible improvement of the eASI’s predictive validity. For example, the rater could indicate in the eASI what the context of the eHealth services is (eg, who is the end user and how is it used). In addition, the rater could indicate if the rating is based on the functionality of the eHealth service itself or on services offered by a remote caregiver. These parameters (context, type of use, and blended care) could be used as covariates for the rating results.

### Online Version of eASI

Currently, an online version of eASI is developed with different functionalities (see Multimedia Appendix 2) [49]. These functionalities could enhance the validity and reliability. In addition, they could contribute to the effectiveness of eASI, regarding analysis and steering. Examples of enhancing functionalities (some of which are already implemented based on the qualitative data elicited in the study) are as follows:

- Using a rating scale instead of dichotomous rating
- Displaying the context of the eHealth service, including the type of use and the involvement of a caregiver
- Adapting the examples, accompanying the items, to the context of the service
- Providing an ontology which clarifies the terminology used in the eASI
- Providing examples of services which score high or low per items of the eASI
- Summarizing rating results and suggesting improvements for the service
- Offering the rater the possibility to provide an overall personal grade for the rated service
- Sharing results among raters

In a future study, we will evaluate if these functionalities further contribute to the reliability and validity.

### Steering eHealth to Greater Effect on Self-Management

The results show that the eASI can analyze eHealth services, but also can provide directions for improvement of eHealth services. While developing eHealth services, developers could bare the items of eASI in mind. The more items are fulfilled, the greater the chance that the eHealth service will be effective in regard to stimulating self-management. However, specific eASI items could be at odds. For instance, when implementing cognitive behavioral therapy (CBT) in an eHealth service, the item “The eHealth service contains game elements” is unconventional. Still, through challenge and development of competencies, games can greatly contribute to long-term interaction. Stimulating behavior (ie, develop new healthy behavior or stop unhealthy behavior) takes time and gaming could stimulate people to use eHealth longer. Thus, we recommend developers not to rigidly adhere to the items of eASI, but incorporate the instrument in a conscious decision-making process, during the design of the service.

These results also show that the eASI has added value in terms of scientific contributions to eHealth evaluations. Greenhalgh and Russell [5] point out that “assumptions, methods, and study designs of experimental science, whilst useful in many contexts, may be ill-suited to the particular challenges of evaluating eHealth programs” (p. 2). They provide an alternative set of guiding principles for eHealth evaluation based on traditions that view evaluation as social practice rather than as scientific testing. In the light of this paper, the eASI facilitates applying the suggested guiding principles related to the creation of interpersonal and analytic space for effective dialog, the consideration of the meso-level contexts (eg, organizations, professional groups), and the consideration of the individuals (eg, clinicians, managers, and service users) through whom the eHealth innovation(s) will be adopted, deployed, and used. Illustratively, the eASI provides a theory-based reference for the dialog between stakeholders, who are involved in the buying (insurers), providing (caregivers), and developing (developers) of eHealth for a variety of end users, for example, people who are overweight or cope with a chronic illness. With the eASI, these stakeholders have a starting point to jointly determine what, on the one hand, can theoretically contribute to the effectiveness of eHealth on the level of the intervention itself (ie, utility, usability, and content). On the other hand, it can help translate eASI rating outcomes to implications for among other insurance companies, care organizations, and patient associations to come to an overall improved eHealth. The eASI can aid decision making in regard to reimbursing and/or providing an eHealth service or not and further development or not. This in the end goes at the benefit of the ehealth user.

When using the eASI, it is important to also consider other instruments, which can contribute to improve the effective application of eHealth, such as HWRI, e-hit, and CONSORT-EHEALTH [6,8,9]. The eASI showed to have multiple unique qualities to be an addition to the domain of eHealth evaluation, that is, a quick, but not dirty way to forecast eHealth effectiveness in regard to self-management. However, other instruments could be more suitable depending on the phase of development (eg, reporting the evaluation or implementation).

### Limitations

This study has a number of limitations. First, the sample size of the study is a major limitation. We were restricted by the amount of services, which on the one hand were trialed in an RCT and, on the other hand were available to rate. However, to compute a correlation the sample size was sufficient. A minimum of 15 observations is recommended [50]. Second, we did not evaluate the RCTs of eHealth services on methodological quality. As a result, it is possible that included studies that found smaller effect sizes actually were more methodologically sound than other included studies. Third, 13 of the 16 studied and available eHealth services were from the Dutch origin. This could be explained as follows. We selected the eHealth service using the Dutch, English, French, or German language to enable rating the services. This diminishes the inclusion of services from the regions Asia, South-America, and Africa. The second explanation is that within the remaining regions (the United States, Australia, and Europe) the Netherlands is the front-runner in the evaluation of eHealth services. Other meta-analyses on eHealth and self-management show that a large number of the services are from the Dutch origin [51,52]. Despite these explanations and as research has found that culture affects the way a person formulates self-management strategies and how a health profession can support these strategies [53], one should recognize the predictive validity of eASI could be different in other countries. Regarding these limitations, it is desirable to continue rating eHealth services, especially from different countries, which are evaluated in high quality RCTs, and further analyze the predictive validity of eASI.

### Conclusions

The eASI is a valid and reliable instrument to predict how effective an eHealth service is in regard to self-management (eg, maintaining diet, performing physical activity, adhering to the low-risk drinking guideline, and controlling corticosteroid). Analysis of an eHealth service with eASI can be conducted quickly and independently of the eHealth user group, which decreases the prerequisite to conduct RCTs. Moreover, the score on eASI and its dimensions utility, usability, and content provide steering how to improve the effectiveness of the service. Although evaluating eHealth is a relatively new and complex field of research, the current results provide an important first step in the development of an instrument to measure the effectiveness of eHealth services supporting self-management. In addition, the eASI can contribute to the dialog regarding to the challenges of evaluating eHealth programs. Specifically, the eASI contributes to “methodological pluralism” suggested to evaluate eHealth by introducing new possibilities to systematically determine and discuss which aspects of eHealth could contribute to effective development, evaluation, and implementation of eHealth for self-management.

## Multimedia Appendix 1

Items of the final version of eASI with 3 dimensions, 11 categories, and 32 items.

## Multimedia Appendix 2

Interfaces of the online version of eASI: rating, summary, and diagnosis.

## Abbreviations

BMI: body mass index
CBT: cognitive behavioral therapy
CONSORT-EHEALTH: Consolidated Standards of Reporting Trials of Electronic and Mobile HEalth Applications and onLine TeleHealth
COPD: chronic obstructive pulmonary disease
eASI: eHealth Analysis and Steering Instrument
HWRI: Health Website Rating Instrument
RCT: randomized controlled trial
