# Supplementary material for: Validity and Reliability of the eHealth Analysis and Steering Instrument
Source: Med 2 0. 2013 Aug 22;2(2):e8. doi: 10.2196/med20.2571 (PMC4085077; doi:10.2196/med20.2571)
Supplement: Supplementary file 1 [file med20_v2i2e8_app1.pdf]

Utility

Item applicable	Yes	No
Insight in health condition		
1 Personal data can be entered in the eHealth service (eg, demographics, personal preferences)		
2 Health data can be entered in the eHealth service (eg, BMI, blood pressure, HbA1c)		
3 The eHealth service presents personal health data schematically (eg, in a graph or table)		
Self-management decision making		
4 The eHealth service describes the possible strategies to cope with a health problem (eg, if someone is overweight he or she can do nothing or exercise more to lose weight)		
5 The eHealth service aids decision making about how to cope with a health problem in agreement with personal preferences (eg, determining the user's preference for exercising more or not)		
Performance of self-management		
6 The eHealth service aids translating chosen coping strategies to a personal goal (eg, my goal is losing 15 kg)		
7 The eHealth service aids setting activities to achieve the personal goal (eg, cycle to work 3 times a week)		
8 The eHealth service monitors the activities performed (eg, keeping track of activities manually or through sensors)		
9 The eHealth service provides advice on how to achieve the set goal (eg, cycle daily at work instead of 3 times a week to lose 15 kg)		

Usability

Item applicable	Yes	No

Interaction

10 The eHealth service provides an overview of the interaction structure and pinpoints the user's current position in the structure (eg, the system provides breadcrumbs navigation)

11 The amount of information the user needs to memorize to be able to use the eHealth service is limited (eg, when logging in the user starts where he or she stopped previously, required information is repeated)

12 The eHealth service can be used on different platforms (eg, smartphone, tablet, PC)

13 The eHealth service offers user support (eg, a helpdesk, help function in the service)

Personalization

14 For each user there is a different version of the eHealth service available (the font size can be changed by the user)

15 The eHealth service attends to the individual's technical skills (eg, literacy, computer skills)

16 The eHealth service attends to the user's individual approach to cope with his or her health problem (eg, expertise on the health problem, level of self-efficacy)

Persuasion

17 The eHealth service contains game elements (eg, it is possible to challenge others, there are different difficulty levels)

18 The eHealth service applies motivating techniques (eg, rewards when a goal is achieved)

19 The eHealth service keeps the user engaged (eg, new content is added to the service)

20 The eHealth service is easy to use (eg, simple to control, fits with daily use)

Content

Item applicable Yes No

Description of health issue

21 The eHealth service specifies which health problem is addressed (eg, being overweight, having alcohol problems)

22 The eHealth service specifies its target group (eg, older adults with physical inabilities, adolescents who are overweight)

Factors of influence

23 The eHealth service addresses personal factors which (partly) cause the health problem (eg, lack of exercise, unhealthy diet)

24 The eHealth service addresses situational factors which (partly) cause the health problem (eg, lack of aid from care organizations, few exercise possibilities in the neighborhood)

Goal of eHealth service

25 The goal of the eHealth service is to engage modifiable factors which cause the health problem (eg, exercising more leads to losing weight)

26 The eHealth service monitors the progress toward the personal goal (eg, the number of kilometer someone walked and still needs to walk to reach the target distance)

Support from the environment

27 The eHealth service provides information about persons and organizations, which can offer support to reach the personal goal (eg, overview of sport associations, caregivers)

28 The eHealth service offers to possibility to contact caregivers (eg, online consult, exchange of information)

29 The eHealth service offers the possibility to call for remote help from persons in the social environment (eg, online peer support, social media)

30 Through the eHealth service, personal and health data can be shared with others (eg, overview of glucose levels can be sent to the caregiver)

Implementation

31 The eHealth service displays the societal support for the service (eg, patient or professional association links its name to the service)

32 The eHealth service provides information on the security of personal data (eg, the user needs to approve the viewing of personal data by the informal and formal caregiver)
