# Supplementary figures and images for: Validity and Reliability of the eHealth Analysis and Steering Instrument
Source: Med 2 0. 2013 Aug 22;2(2):e8. doi: 10.2196/med20.2571 (PMC4085077; doi:10.2196/med20.2571)

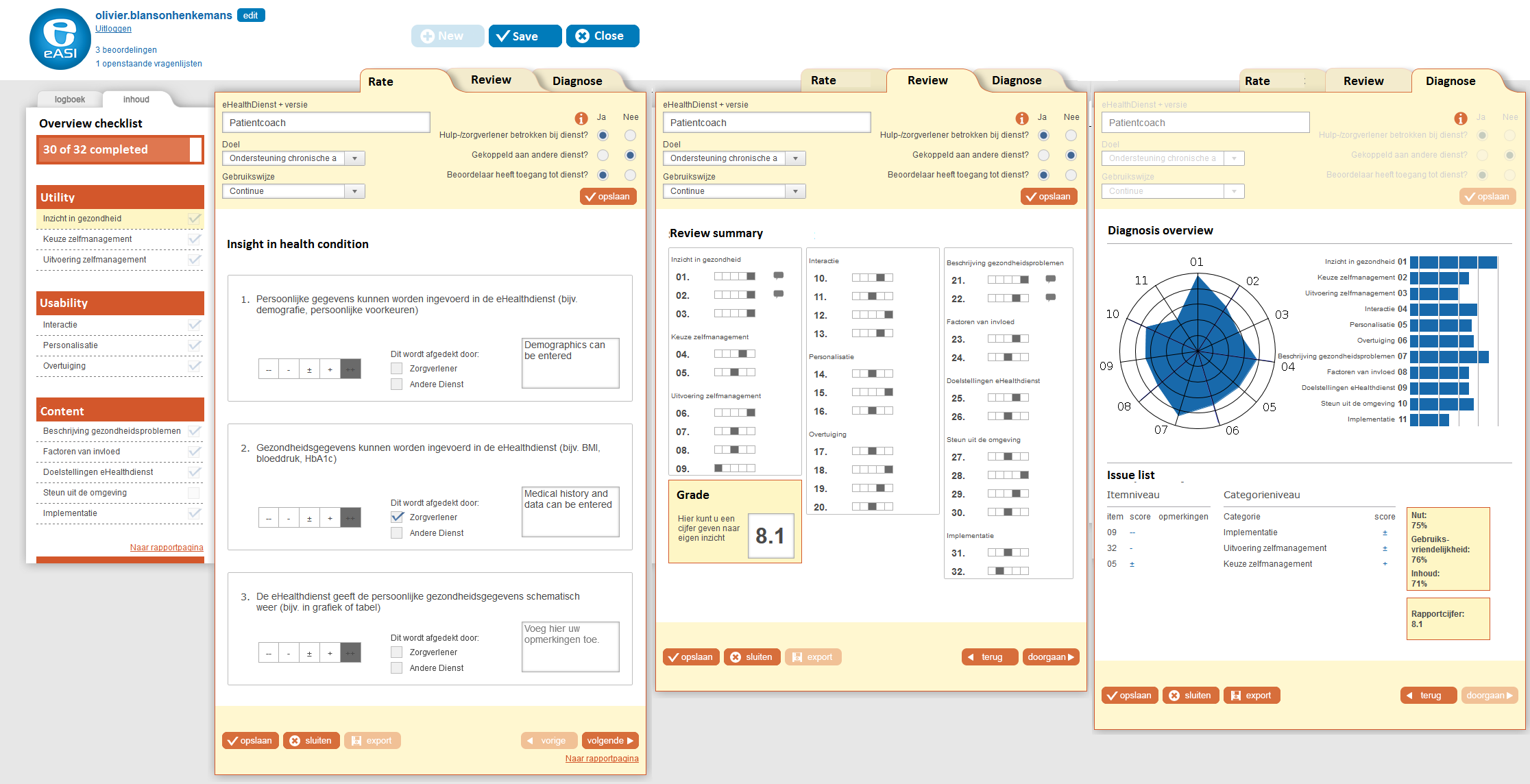

Supplement: Supplementary file 2 [file med20_v2i2e8_app2.png]
